# Study of the cytotoxic effects of 2,5-diaziridinyl-3,6-dimethyl-1,4-benzoquinone (MeDZQ) in mouse hepatoma cells

**DOI:** 10.17179/excli2016-783

**Published:** 2017-03-06

**Authors:** Rasa Jarasiene-Burinskaja, Milda Alksne, Violeta Bartuskiene, Violeta Voisniene, Jaroslav Burinskij, Narimantas Cenas, Virginija Bukelskiene

**Affiliations:** 1 Department of Biological Models, Institute of Biochemistry, Life Sciences Center, Vilnius University, Sauletekio av. 7, LT-10223 Vilnius, Lithuania; 2 Department of Anatomy, Histology and Anthropology, Faculty of Medicine, Vilnius University, M. K. Ciurlionio str. 21, LT-03101 Vilnius, Lithuania; 3 Department of Chemistry and Bioengineering, Vilnius Gediminas Technical University, Sauletekio av. 11, LT-10223 Vilnius, Lithuania; 4 Department of Xenobiotics Biochemistry, Institute of Biochemistry, Life Sciences Center, Vilnius University, Sauletekio av. 7, LT-10223 Vilnius, Lithuania

**Keywords:** aziridinylbenzoquinones, cytotoxicity, cell signaling, cell death, MeDZQ

## Abstract

A number of quinones have been shown to be efficient anticancer agents. However, some mechanisms of their action, in particular cell signaling are not well understood. The aim of this study was to partly fill this gap by characterizing the mode of cytotoxicity of 2,5-diaziridinyl-3,6-dimethyl-1,4-benzoquinone (MeDZQ) in malignant mouse hepatoma cells (MH-22A) with regard to the expression and activation of main molecules in MAPK cell signaling pathway. The study revealed unequal roles of MAP kinases in MeDZQ-induced cell death: the compound did not induce significant changes in ERK expression or its phosphorylation; JNK appeared to be responsible for cell survival, however, p38 kinase was shown to be involved in cell death. In order to assess the enzymatic activation mechanisms responsible for the action of MeDZQ, we have also found that the antioxidant *N,N'*-diphenyl-*p-*phenylene diamine, the iron-chelating agent desferrioxamine, and DT-diaphorase inhibitor, dicoumarol, partly protected the cells from MeDZQ cytotoxicity. It points to parallel oxidative stress and bioreductive alkylation modes of the cytotoxicity of MeDZQ.

## Introduction

Quinones are among the most investigated anticancer agents which have been studied for many years (Lown, 1983[14]; Loadman et al., 2000[13]). There are several modes of their action, among which the most important are:

1) oxidative stress-type cytotoxicity arising from the ability of quinones to form free radicals, which may further undergo redox cycling in aerobic conditions (Cassagnes et al., 2015[9]) thus forming reactive oxygen species (ROS) which provoke quinone-induced apoptotic (Ollinger and Kagedal, 2002[20]; Park et al., 2011[21]) and necrotic (Nemeikaite-Ceniene et al., 2005[18]) cell death; 

2) quinone intercalation with DNA (Sugiura et al., 1990[28]) and/or topoisomerase inhibition, which is mainly characteristic for anthracyclines (Chen and Eastmond, 1995[10]); and 

3) bioreductive activation of aziridinyl-substituted quinones (Sarlauskas et al., 2015[22]). In this case, their net two-electron reduction catalyzed mainly by NAD(P)H: quinone oxidoreductase (NQO1, DT-diaphorase) leads to the formation of corresponding hydroquinones that alkylate DNA more readily than the parent quinones (Begleiter and Blair, 1984[3]; Morgan et al., 1997[17]; Bolton et al., 2002[5]; Siegel et al., 2012[27]; Sarlauskas et al., 2015[22]). 

**Figure 1:**
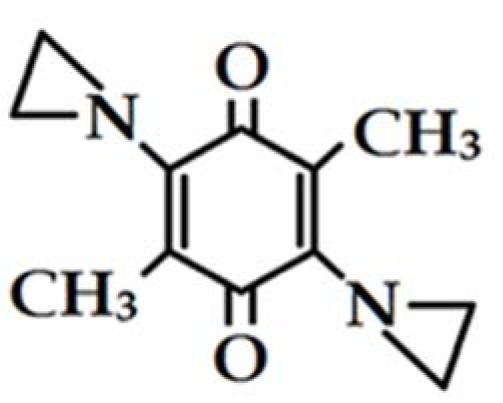
Structure of 2,5-aziridinyl-3,6-dimethyl-1,4-benzoquinone (MeDZQ).

One of potential antitumor quinones is 2,5-diaziridinyl-3,6-dimethyl-1,4-benzoquinone (MeDZQ) (Figure 1(Fig. 1)). Its redox properties, *e.g., *the single-electron reduction potential (*E*^1^_7_, or the redox potential of quinone/semiquinone couple, -0.23 V), and reactivity towards the main enzymes responsible for its single- and two-electron reduction, respectively, NADPH:cytochrome P-450 reductase and NQO1, are similar to those of its aziridinyl-unsubstituted analogue, tetramethyl-1,4-benzoquinone (duroquinone, DQ, *E*^1^_7_ = -0.26 V (Miliukiene et al., 2014[16])). 

Previous studies have shown that the reduction of aziridinyl-substituted quinones can regulate the activation of mitogen-activated protein kinases (MAPK) (Seanor et al., 2003[24]; Park et al., 2011[21]). In mammals, there are three main subfamilies of MAPKs: extracellular signal-regulated kinases (ERKs), c-Jun N-terminal kinases (JNKs) and p38 isoforms (p38-MAPKs) (Wada and Penninger, 2004[30]; Huang et al., 2010[12]). It has been shown that ERKs are mostly important for cell survival (Wada and Penninger, 2004[30]), whereas JNK and p38 kinases respond to a variety of stress signals (ionizing radiation, oxidative stress, inflammatory cytokines) and are known as the stress-activated protein kinases (SAPKs). They are shown to be involved in cell death processes (Wada and Penninger, 2004[30]; Huang et al., 2010[12]). However, the role of DNA damage in the activation of SAPKs is complex; it is known that the immediate cell response is due to receptor activation whereas the late response involves DNA damage (Batista et al., 2009[2]). Therefore, the elucidation of the signaling pathways involved in quinone-induced apoptotic cell death may be useful in understanding of the modes of action of chemotherapeutical agents and their further development.

## Materials and Methods

### Chemicals and reagents

MeDZQ was a generous gift by Dr. Jonas Sarlauskas (Department of Xenobiotics Biochemistry, Institute of Biochemistry, Life Sciences Center, Vilnius University). It was synthesized according to a previously published procedure (Cameron and Gilles, 1968[7]; Winski et al., 1998[31]). Fetal bovine serum (FBS) was obtained from Life Technologies (USA), penicillin-streptomycin, trypsin and EDTA were purchased from Biological Industries (Israel). Dulbecco's modified Eagle medium (DMEM), acridine orange/ethidium bromide (AO/EB) mixture, 3-(4,5-dimethylthiazol-2-yl)-2,5-diphenyltetrazolium bromide (MTT), dicumarol (DIC), *N,N'*-diphenyl-*p*-phenylenediamine (DPPD), desferrioxamine (DESF), MAPK inhibitors PD098059, SP600125 and SB203580 were obtained from Sigma-Aldrich (USA). Stock solutions (20 mM) of all the inhibitors were prepared in dimethyl sulfoxide (DMSO). Anti-phospho-p38, anti-ERK and anti-JNK antibodies were purchased from Cell Signaling Technology (USA). Monoclonal antibodies for p38, phospho-ERK, and phospho-JNK detection were obtained from BD Biosciences (USA). Secondary goat anti-mouse and goat anti-rabbit antibodies (both conjugated with horseradish peroxidase) were purchased from Abcam (UK). Low melting-point agarose (LMPA) was purchased from Carl Roth (Germany), and standard agarose - from Thermo Fisher Scientific (USA).

### Cell culture and treatments

To investigate MeDZQ toxicity, we used malignant mouse hepatoma cell line (MH-22A) (Shvemberger and Alexandrova, 2000[26]). The cells were grown in DMEM supplemented with 10 % of FBS, penicillin (100 U/ml), and streptomycin (100 μg/ml). The cells were maintained at 37° C in a humidified atmosphere with 5 % CO_2_, and passaged twice a week by detaching the cells with a 0.25 % trypsin/EDTA solution. For cytotoxicity testing, MH-22A cells were seeded at a density of 3.0×10^5^ cells/ml in 96-well flat bottom plates (Sigma-Aldrich, USA) (100 µl/well) 24 h before the start of the experiment. After reaching 60-80 % confluency, the cells were treated with MeDZQ, dissolved in DMSO (factor of dilution 1:2000).

Inhibitors (DIC (20 µM), DPPD (2 µM), DESF (300 µM), PD098059 (20 µM), SP600125 (20 µM) and SB203580 (20 µM)) were added to the culture 30 min prior to MeDZQ exposure. All inhibitors were prepared in DMSO, the final concentration of DMSO in the cell growth medium never exceeded 0.1 %. A corresponding amount of DMSO was added to the culture medium as a second control (first control - intact cells).

### Cell viability analysis

After 24 h incubation with different concentrations of MeDZQ, the cells were washed with phosphate-buffered saline and treated with MTT solution (final concentration, 0.2 mg/ml) for 1 h at 37° C. MTT is reduced to purple formazan only in living cells. Afterwards, it was dissolved in ethyl alcohol (100 µl) and the absorbance was measured at 570 nm using a microplate spectrophotometer Varioskan Flash (Thermo Scientific). The absorbance corresponds to the fraction of viable cells in the sample. The results were expressed as relative viability,* i.e. *average of ratio: treated cells/control (intact) cells. The concentration of MeDZQ that killed 50 % of the cells in the test culture was considered LC_50_.

### Study of cell death

Cell death after MeDZQ exposure was assessed using two methods.

The first one was microscopic cell analysis after staining with AO/EB mixture (Mercille and Massie, 1994[15]). In short, the cells were grown for 24 h at MeDZQ LC50, then collected and 20 µl of cell suspension were mixed with AO/EB (3 µl) and analyzed using a fluorescence microscope (Olympus IX51). In each sample, 3x100 cells were calculated to determine the numbers of viable, viable apoptotic, nonviable apoptotic, necrotic and chromatin-free cells.

The second method used was flow cytometry (Guava easyCyte 8HT, Millipore). After quinone treatment, 200 µl of cell suspension (100-500 cells/µl) was mixed with 2 µl (0.1 mg/ml) AO/EB, incubated for 5 min, and then flow cytometry assay was performed.

### Western blot analysis

The effect of MeDZQ on MAP kinases expression and phosphorylation was evaluated using Western blot. Cells were lysed with ice-cold buffer (10 mM Tris HCl pH 7.4, 50 mM NaCl, 5 mM EDTA, 50 mM NaF, 0.1 % BSA, 1 % Triton X-100, 1 mM PMSF, 2 mM Na_3_VO_4_, 20 µg/ml aprotinin, pH 7.2). Insoluble materials were removed by centrifugation at 14 000 rpm for 15 min at 0-4° C. The remaining supernatant was then mixed with equal parts of 4×SDS sample buffer. Protein amounts were estimated using Bradford protein assay. Subsequently, SDS-PAGE (10 % acrylamide gels) was performed, and separated proteins were transferred onto PVDF membranes. The blots were blocked with blocking buffer, 3 % low fat milk in TBST (4 mM Tris HCl, 1 mM Tris base saline, 0.1 % Tween 20, 154 mM NaCl), for 30 min at room temperature. The membranes were washed by TBST (3 x 5 min), probed first with primary antibody in 5 % low fat milk in TBST for 24 h at 4° C, and then with the secondary antibody in the same 5 % low fat milk in TBST for 1 h at room temperature. After the incubation, membranes were washed by TBST (3 × 5 min). Proteins were visualized using an Amersham ECL system.

### Statistical analysis

Statistical analysis was performed using the Student's t-test (Excel 2013, Microsoft). Data is presented as a mean ± standard error of at least 3 independent experiments. Differences were considered statistically significant at p < 0.05.

## Results and Discussion

**Figure 2:**
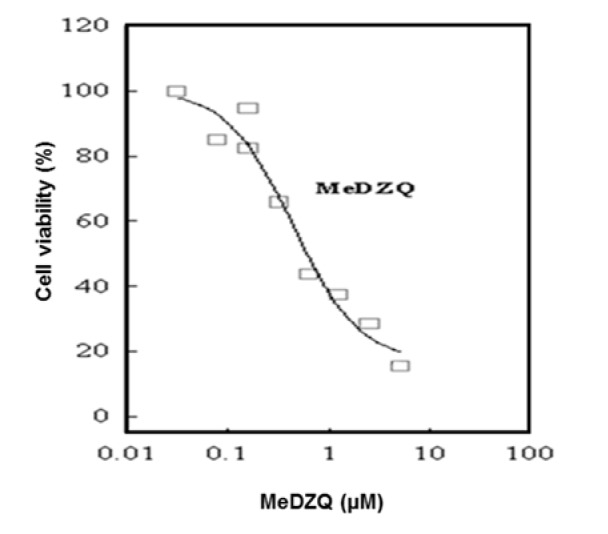
Cell viability decrease depending on the concentration of MeDZQ after a 24 h exposure (n* ≥ *3); LC_50_ of MeDZQ was found to be 0.31 ± 0.1 µΜ.

First, the LC_50 _of MeDZQ was assessed - it was 0.31 ± 0.1 µΜ after 24 h of exposure (Figure 2(Fig. 2)). This MeDZQ concentration was used in all subsequent experiments.

Before investigating the mechanisms of activity, the mode of cell death induced by MeDZQ was determined.

**Figure 3:**
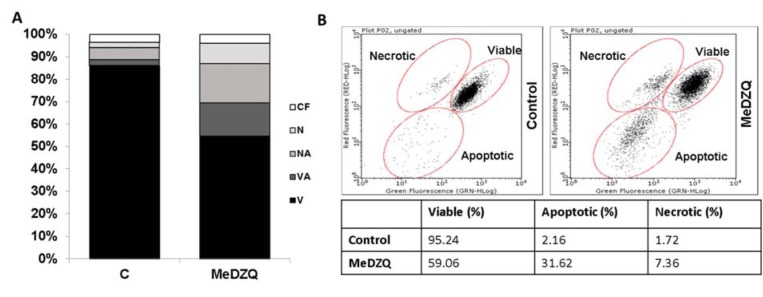
Cell viability analysis after 24 h of MeDZQ (0.31 µM) treatment (n* ≥ *3): (A) - cells stained with AO/EB dye mixture (V - viable; VA - viable apoptotic; NA - non-viable apoptotic; N - necrotic; CF - chromatin-free); (B) - cell death analysis by flow cytometry (n* ≥ *3); percentage points of viable, apoptotic and necrotic cell distribution.

It was carried out using fluorescence microscopy and flow cytometry. There are studies reporting that quinones induce apoptotic cell death (Sun and Ross, 1996[29]; Fourie et al., 2002[11]; Park et al., 2011[21]). Our results confirmed this fact showing that MeDZQ at LC_50_ induced apoptosis: AO/EB staining demonstrated 17 % of nonviable apoptotic and 15 % viable apoptotic cells (Figure 3A(Fig. 3)). A similar tendency was observed in the flow cytometry analysis. The data in Figure 3B(Fig. 3) shows a large number (more than 30 %) of apoptotic cells appearing after MeDZQ treatment.

**Figure 4:**
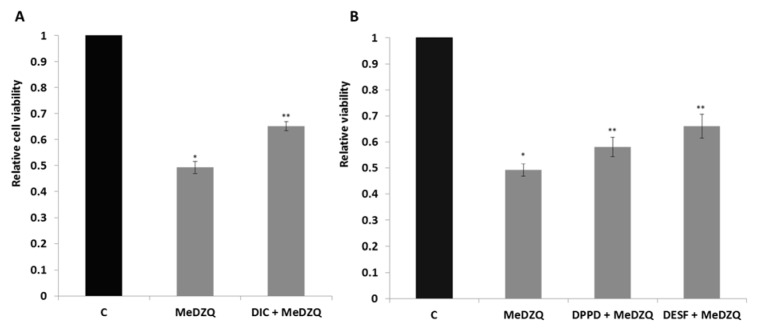
MH-22A cell viability after the treatment with (A) - MeDZQ together with NQO1 inhibitor DIC and (B) - antioxidants: DPPD and DESF. The results were registered using MTT assay and are presented as the average of relative viability, calculated from at least 3 independent experiments, ± SD. *Statistically reliable values between the viability of control (C) and MeDZQ treated cells (p < 0.05). **Statistically significant differences between cells treated solely with MeDZQ and DIC+MeDZQ, DPPD+MeDZQ, DESF+MeDZQ (p < 0.05).

One of the main mechanisms responsible for aziridinyl-benzoquinone-induced cell death is their two-electron reduction to corresponding hydroquinones by NAD(P)H: quinone oxidoreductase (NQO1). The activity of NQO1 in MH-22A cells is 79.5 nmol cytochrome *c *reduced × mg protein^-1 ^× min^-1^ (Nemeikaite-Ceniene et al., 2015[19]). In order to evaluate the role of NQO1 in MeDZQ-induced cell death, we used DIC, a well-known NQO1 inhibitor (Scott et al., 2011[23]) which competes with NAD(P)H for binding to the active center of the enzyme. The protective effect of DIC shows that NQO1 significantly contributes to MeDZQ-induced cytotoxicity (Figure 4A(Fig. 4)).

In order to assess other causes of cell death, the role of oxidative stress was evaluated. For this purpose, we examined the effects of antioxidant DPPD and Fe-ion chelator DESF, because both of them were shown to be efficient in other studies (Shimoni et al., 1994[25]; Nemeikaite-Ceniene et al., 2005[18]). Our results confirmed that DPPD and DESF protected the cells against the effect of MeDZQ (Figure 4B(Fig. 4)). It means that oxidative stress is a parallel mechanism of MeDZQ cytotoxicity.

Among the many signaling pathways that respond to stress, MAPK family members (ERK, JNK, p38) are crucial for the cell fate. Two of them, JNK and p38, are known as SAPKs. There is evidence that following quinone exposure, some SAPKs take part in apoptosis induction (Park et al, 2011[21]). 

**Figure 5:**
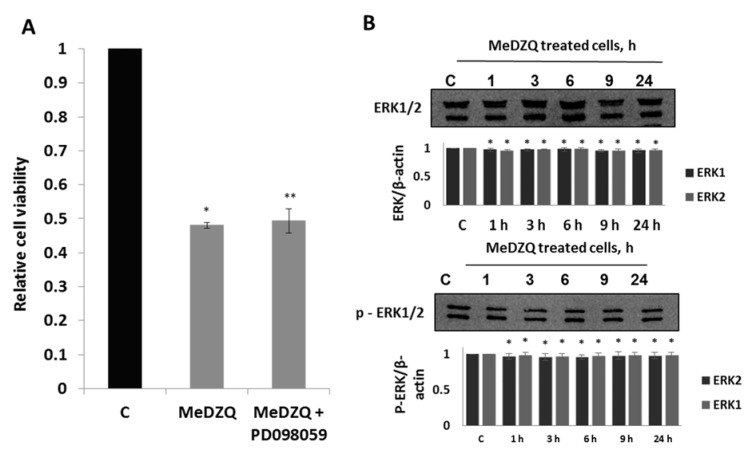
(A) - the effect of ERK kinase inhibitor on MH-22A cell viability at 24 h after MeDZQ and MeDZQ+PD098059 exposure, the results were registered using MTT assay and are presented as the average of relative viability, calculated from at least 3 independent experiments, ± SD. * Statistically significant differences between the viability of control (C) and MeDZQ treated cells (p < 0.05). **Statistically significant differences between the viability of cells treated solely with MeDZQ and MeDZQ+PD098059 (p < 0.05). (B) - representative immunoblot images showing the ERK1/2 and phospho-ERK1/2 protein expression in MH-22A cells after MeDZQ exposure; β-actin was used as a loading control. The relative level of the ERK1/2 and phospho-ERK1/2 protein expression was normalized to β-actin and was expressed as the ratio of ERK1/2 /β-actin and phospho-ERK1/2 /β-actin as indicated at the bottom of the image; *p < 0.05, (n ≥ 3).

It is known that ERK signaling pathway is involved in proliferation, differentiation and survival, however, there is data showing that ERK may participate in the activation of apoptotic cell death (Cagnol and Chambard, 2010[6]). We assessed the role of ERK by using its indirect inhibitor PD098059 which acts on ERK by inhibiting MEK1/2 activity (Alessi et al., 1995[1]). We have found that in our model of malignant MH-22A cells, the ERK inhibitor did not affect cell viability in the presence of MeDZQ. The changes in ERK expression and activation were also not detected (Figure 5A, B(Fig. 5)). Apparently, in MeDZQ induced MH-22A cell death, ERK is not relevant.

**Figure 6:**
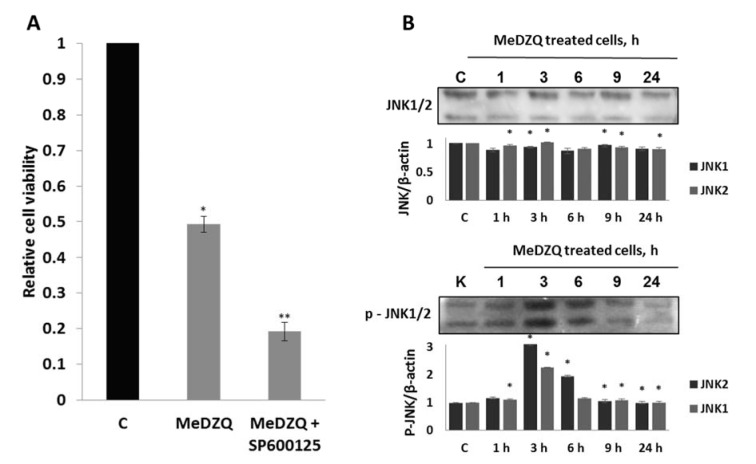
(A) - the effect of JNK inhibitor on MH-22A cell viability at 24 h after MeDZQ and MeDZQ+SP600125 exposure; MTT test absorbance averages from at least 3 independent experiments ± SD. *Statistically significant differences between the viability of control (C) and MeDZQ treated cells (p < 0.05). **Statistically significant differences between the viability of cells treated solely with MeDZQ and MeDZQ+SP600125 (p < 0.05). (B) - representative immunoblot images showing the JNK1/2 and phospho-JNK1/2 protein expression in MH-22A cells after MeDZQ exposure; β-actin was used as a loading control. The relative level of the JNK 1/2 and phospho-JNK1/2 protein expression was normalized to β-actin and was expressed as the ratio of JNK1/2 /β-actin and phospho-JNK1/2 /β-actin as indicated at the bottom of the image; *p < 0.05, (n ≥ 3).

In contrast, the inhibitor of JNK, SP600125, which directly inhibits signal transduction, thus disabling the activation of one of the main downstream targets in the nucleus c-Jun (Bennett et al., 2001[4]; Cargnello and Roux, 2011[8]), potentiated the cytotoxicity of MeDZQ (Figure 6A(Fig. 6)). However, the total amount of JNK in MH-22A cells did not change during 1-24 h incubation with MeDZQ. In contrast, the amount of activated (phosphorylated) JNK increased up to 6 h exposure to MeDZQ and then started to gradually decrease (Figure 6B(Fig. 6)). The effect shows the early role of this kinase which looks like antiapoptotic in this case.

**Figure 7:**
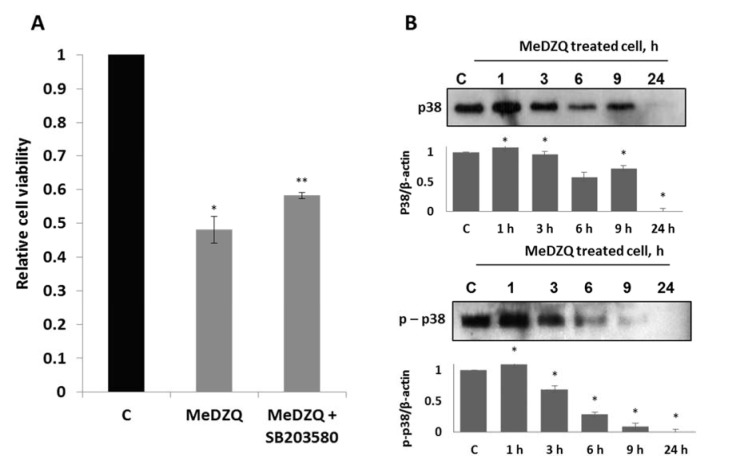
(A) - the effect of p38 kinase inhibitor on MH-22A cell viability at 24 h after MeDZQ and MeDZQ+SB203580 exposure; MTT test absorbance averages calculated from at least 3 independent experiments ± SD. *Statistically significant differences between the viability of control (C) and MeDZQ treated cells (p < 0.05). **Statistically significant differences between the viability of cells treated solely with MeDZQ and MeDZQ+SB203580 (p < 0.05). (B) - representative immunoblot images showing the protein levels of p38 and phospho-p38 after the MH-22A cell exposure to MeDZQ; β-actin was used as a loading control. The relative level of the p38 and phospho-p38 protein expression was normalized to β-actin and was expressed as the ratio of p38/β-actin and phospho-p38/β-actin as indicated at the bottom of the image; *p < 0.05, (n ≥ 3).

In terms of p38, its inhibitor SB203580 partly protected cells from MeDZQ-induced cell death (Figure 7A(Fig. 7)). It suggests that p38 kinase contributes to cell death in this case. 

According to our data, during the first 3 h after the exposure, total amount of p38 protein was comparable (Figure 7B(Fig. 7)). However, following the exposure to MeDZQ after 24 h, the amount of protein decreased significantly. The levels of phospho-p38 increased after 1 h of exposure, but decreased after 3 h and disappeared after 24-hour exposure. Therefore, contrary to JNK, p38 showed proapoptotic activity upon MeDZQ effect. This is contradictory to literature findings that cells with a large amount of NQO1 do not influence aziridinyl-benzoquinone induced apoptotic cell death via p38 kinase (Park et al., 2011[21]).

The results show that the LC_50_ for the aziridinyl-benzoquinone MeDZQ was 0.31 ± 0.1 µM in MH-22A cell culture. MeDZQ at LC_50_ caused apoptotic cell death. NQO1 inhibitor DIC increased viability of MH-22A cells following the MeDZQ exposure. The significant protective effect of DIC has demonstrated that NQO1 significantly contributes to MeDZQ-induced cytotoxicity. Furthermore, the antioxidants DPPD and DESF protected the cells against the action of MeDZQ, which showed the involvement of oxidative stress in the quinone-induced cell death. MAP kinase study revealed unequal role in MeDZQ-induced cell death: the test compound did not induce significant changes in ERK expression, nor in its phosphorylation; JNK signaling appeared responsible for cell survival, however, p38 kinase might be involved in cell death.

## Acknowledgements

This research was funded by the European Social Fund under Global Grant Measure No. VP1-3.1-ŠMM-07-K01-103 (Research Council of Lithuania).

## Conflict of interest

The authors declare no conflict of interest.
